# Patterns of population structure at microsatellite and mitochondrial DNA markers in the franciscana dolphin (*Pontoporia blainvillei*)

**DOI:** 10.1002/ece3.2596

**Published:** 2016-11-17

**Authors:** María Constanza Gariboldi, Juan Ignacio Túnez, Mauricio Failla, Marta Hevia, María Victoria Panebianco, María Natalia Paso Viola, Alfredo Daniel Vitullo, Humberto Luis Cappozzo

**Affiliations:** ^1^ Centro de Estudios Biomédicos, Biotecnológicos, Ambientales y Diagnóstico (CEBBAD)Universidad MaimónidesCiudad Autónoma de Buenos AiresArgentina; ^2^ Consejo Nacional de Investigaciones Científicas y Técnicas (CONICET)Ciudad Autónoma de Buenos AiresArgentina; ^3^ Grupo de Estudios en Ecología de MamíferosDepartamento de Ciencias BásicasUniversidad Nacional de LujánLujánArgentina; ^4^ Fundación CethusBuenos AiresArgentina; ^5^ Laboratorio de EcologíaComportamiento y Mamíferos MarinosMuseo Argentino de Ciencias Naturales “Bernardino Rivadavia”Ciudad Autónoma de Buenos AiresArgentina; ^6^ Laboratorio de Ecología y Conservación de Vida SilvestreCentro Austral de Investigaciones Científicas (CADIC)UshuaiaArgentina; ^7^ Fundación de Historia Natural Félix de AzaraUniversidad MaimónidesCiudad Autónoma de Buenos AiresArgentina

**Keywords:** microsatellites, mitochondrial DNA, *Pontoporia blainvillei*, population structure

## Abstract

The franciscana dolphin, *Pontorporia blainvillei*, is an endemic cetacean of the Atlantic coast of South America. Its coastal distribution and restricted movement patterns make this species vulnerable to anthropogenic factors, particularly to incidental bycatch. We used mitochondrial DNA control region sequences, 10 microsatellites, and sex data to investigate the population structure of the franciscana dolphin from a previously established management area, which includes the southern edge of its geographic range. *F*‐statistics and Bayesian cluster analyses revealed the existence of three genetically distinct populations. Based on the microsatellite loci, similar levels of genetic variability were found in the area; 13 private alleles were found in Monte Hermoso, but none in Claromecó. When considering the mitochondrial DNA control region sequences, lower levels of genetic diversity were found in Monte Hermoso, when compared to the other localities. Low levels of gene flow were found between most localities. Additionally, no evidence of isolation by distance nor sex‐biased dispersal was detected in the study area. In view of these results showing that populations from Necochea/Claromecó, Monte Hermoso, and Río Negro were found to be genetically distinct and the available genetic information for the species previously published, Argentina would comprise five distinct populations: Samborombón West/Samborombón South, Cabo San Antonio/Buenos Aires East, Necochea/Claromecó/Buenos Aires Southwest, Monte Hermoso, and Río Negro. In order to ensure the long‐term survival of the franciscana dolphin, management and conservation strategies should be developed considering each of these populations as different management units.

## 1 Introduction

Along the distribution range of a species, individuals may conform to different populations with different degrees of isolation in response to environmental and/or behavioral factors (Adams & Rosel, 2006; Hoelzel, Dahlheim, & Stern, 1998; Lázaro, Lessa, & Hamilton, 2004; Lessios, 2008; Sellas, Wells, & Rosel, 2005). Commonly, molecular genetics analyses of population structure are used to understand the dynamic of these populations and to facilitate the formulation of effective conservation strategies. Mitochondrial DNA (mtDNA) and microsatellite markers are the most popular markers used for this purpose (e.g., Baker et al., 1998; Costa Urrutia, Abud, Secchi, & Lessa, 2012; Escorza Treviño & Dizon, 2000; Lyrholm, Leimar, Johanneson, & Gyllensten, 1999; Méndez, Rosenbaum, Subramaniam, Yackulic, & Bordino, 2010; Natoli, Birkun, Aguilar, Lopez, & Hoelzel, 2005; Natoli, Peddemors, & Hoelzel, 2008; Pope, Sharp, & Moritz, 1996; Tonione, Johnson, & Routman, 2011). Due to the easy collection, inheritance, lack of recombination, and fast rates of base substitution, mtDNA has been extensively used as a marker in phylogeographic studies (Avise, 1994; Dowling, Moritz, Palmer, & Rieseberg, 1996; Pope et al., 1996; Tonione et al., 2011). However, due to its maternal inheritance, population studies based only on this locus may be biased to female‐mediated processes (Avise, 1994; Pope et al., 1996; Zhang & Hweitt, 2003). On the other hand, microsatellites are present widely throughout the euchromatic portion of genomes, are highly polymorphic and apparently neutral, and biparentally inherited. Their introduction in population genetic studies improved our ability to assess genetic diversity, parentage and relatedness, fine‐scale population structure, and recent population history. However, genealogical patterns of relationships cannot be deduced because of the ambiguity of the ancestral information that they contain (Pope et al., 1996; Zhang & Hweitt, 2003). Therefore, incorporating both types of markers may enhance our understanding on the historical and present demographic events that shape the population structure of a species.

The franciscana, *Pontoporia blainvillei*, is a small dolphin that inhabits coastal waters and estuaries of the South Atlantic coast of America. Its distribution, restricted within the 30 m isobaths from the coast, is comprised between Itanúas (18°25′ S), Brazil, and Golfo San Matías (41°10′ S), Argentina (Crespo, 2009; Crespo, Pedraza, Grandi, Dans, & Garaffo, 2010; Pinedo, Praderi, & Brownell, 1989; Secchi, Danilewicz, & Ott, 2003). Due to its reduced movement patterns and to anthropogenic impacts, it is the most threatened small cetacean on the southwestern Atlantic Ocean; it is particularly susceptible to high incidental bycatch mortality throughout its distribution (Bordino, Mackay, Werner, Northridge, & Read, 2013; Bordino, Wells, & Stamper, 2008; Bordino et al., 2002; Cappozzo et al., 2007; Crespo, Corcuera, & Lopez Cazorla, 1994; Crespo et al., 2010; Di Beneditto, 2003; Negri, Denuncio, Panebianco, & Cappozzo, 2012; Secchi, 2010; Secchi et al., 1997, 2003). Based on a projected species abundance decline of more than 30% over three generations (36 years; Taylor, Chivers, Larese, & Perrin, 2007), the franciscana dolphin was classified as “Vulnerable” by the International Union for Conservation of Nature (IUCN) in 2008 (Reeves et al., 2012).

**Figure 1:**
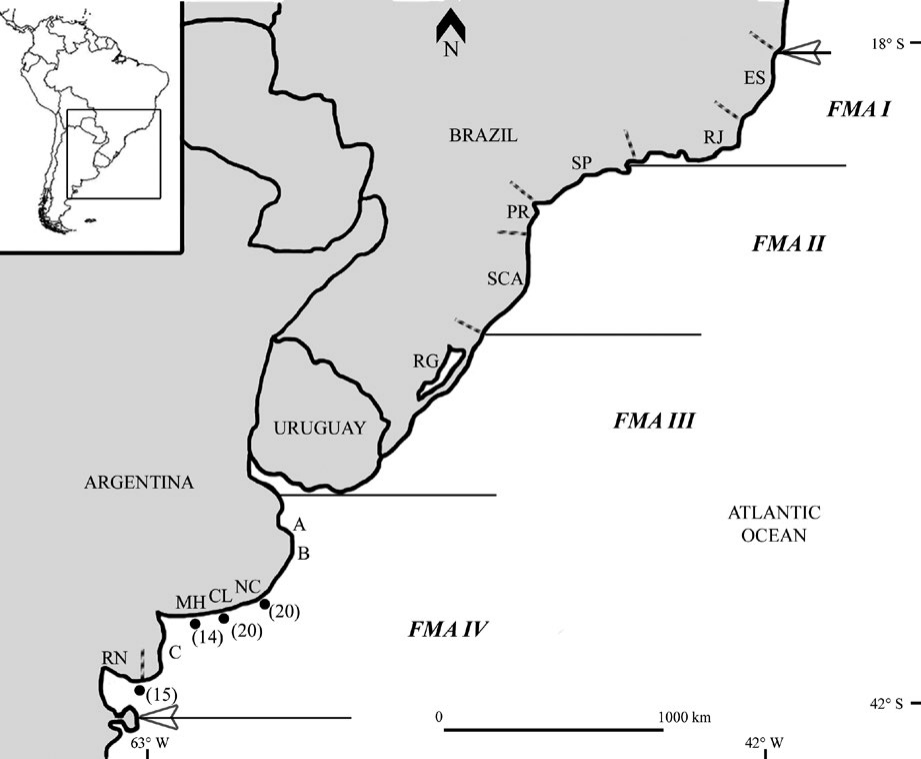
Franciscana Management Areas (FMAs) and sampled sites. Previously proposed FMAs (FMA I‐IV) (Secchi et al., 2003) are delineated with solid lines. The number of samples is shown between brackets. State and Province limits are delineated with dashed lines. Letters A, B, and C correspond to Samborombón West (SW)/Samborombón South (SS), Cabo San Antonio (CSA)/Buenos Aires East (BAE), and Buenos Aires Southwest (BASW) sampled sites, respectively (Méndez, Rosenbaum, Subramaniam, et al. 2010; Méndez, Rosenbaum, Wells, et al., 2010). ES, Espírito Santo; RJ, Rio de Janeiro; SP, São Paulo; PR, Paraná; SCA, Santa Catarina; RG, Rio Grande do Sul; NC, Necochea; CL, Claromecó; MH, Monte Hermoso; RN, Río Negro

Considering the available data on the franciscana dolphin geographic distribution, abundance, reproductive strategies, age at sexual maturity, growth parameters, and mtDNA analyses, among other characteristics, Secchi et al. (2003) divided the species distribution range into four different areas called Franciscana Management Areas (FMAs): FMA I (from Espíritu Santo to Rio de Janeiro, Brazil), FMA II (from São Paulo to Santa Catarina, Brazil), FMA III (from Rio Grande do Sul, Brazil, to Uruguay), and FMA IV (Argentina) (Figure 1). Subsequent studies confirmed Secchi et al.'s subdivision and further suggested the existence of genetically differentiated populations within the FMAs (Costa Urrutia et al., 2012; Cunha et al., 2014; Gariboldi et al., 2015; Méndez, Rosenbaum, & Bordino, 2008; Méndez, Rosenbaum, Subramaniam, et al., 2010; Negri, 2011; Valsecchi & Zanelatto, 2003). Particularly, based on mtDNA and microsatellite data, the FMA IV would comprise four populations: (1) Samborombón West (SW)/Samborombón South (SS), (2) Cabo San Antonio (CSA)/Buenos Aires East (BAE), (3) Monte Hermoso (MH), and (4) Necochea (NC)/Claromecó (CL)/Buenos Aires Southwest (BASW)/Río Negro (RN) (Gariboldi et al., 2015; Lázaro et al., 2004; Méndez et al., 2008; Méndez, Rosenbaum, Subramaniam, et al., 2010) (Figure 1).

Following Gariboldi's et al. (2015) suggestion of increasing the number of loci and samples to acquire a better understanding of the historical and present processes that shape the genetic structure of the franciscana dolphin from the south FMA IV, in this study we combine microsatellite and mtDNA markers to further analyze the franciscana population structure at the southern edge of the species distribution.

## 2 Materials and methods

### 2.1 Tissue sample collection and DNA extraction

From 2000 to 2013, we collected tissue samples from 71 incidentally entangled and/or stranded franciscana dolphins from four localities along the coastal area between Necochea (NC) and Río Negro (RN) (from Viedma to Punta Bermeja), Argentina (Figure 1). Sampling permits were issued by Dirección de Fauna Silvestre (Río Negro Province) and Dirección Provincial de Fiscalización y Uso Agropecuario de los Recursos Naturales (Buenos Aires Province), Argentina. Since three individuals were by‐caught simultaneously in this study, and considering that franciscana dolphins would travel in groups comprised in part of close relatives (Costa Urrutia et al., 2012; Méndez, Rosenbaum, Wells, Stamper, & Bordino, 2010; Valsecchi & Zanelatto, 2003), we analyzed only one of those individuals to reduce a potential bias in our analyses. Furthermore, all incidentally entangled or stranded franciscana dolphins used in this study were collected within each locality in sampling events that took place with at least one month of separation between each other. Tissue samples were preserved in 20% dimethyl sulfoxide (DMSO) and/or 96% ethanol.

### 2.2 Laboratory analyses

Total DNA was extracted from tissue samples using a proteinase K digestion, extraction of proteins with a phenol–chloroform method, and alcohol precipitation of DNA (Sambrook, Fritsch, & Maniatis, 1989).

#### 2.2.1 Sex determination

In order to determine the sex of individuals, a multiplex polymerase chain reaction (PCR) that targets ZFX and SRY genes was performed (Rosel, 2003). PCR amplifications were performed in a 20 μl reaction volume consisting of 5 μg/ml of template DNA, Buffer 1X (Promega), 0.2 mmol/L of dNTPS, a variable concentration of each primer (Table S1), 1.5 mmol/L of MgCl2, and 1.25 units of GoTaq polymerase (Promega). PCR cycling profile consisted on an initial denaturation at 94°C for 30 s, followed by 35 cycles of denaturation at 94°C for 30 s, annealing at 51°C for 40 s and polymerase extension at 72°C for 40 s, and a final extension at 72°C for 1 min. As positive controls, individuals with known sex confirmed through necropsy were included.

#### 2.2.2 Microsatellite loci

Ten microsatellite loci developed for other cetacean species (Table S2) and already utilized for franciscana dolphins (Costa Urrutia et al., 2012; Méndez, Rosenbaum, Subramaniam, et al., 2010) were amplified. All forward primers were modified with a fluorescent dye. Final concentrations used in PCRs of 25 μl volume were as follows: 5 μg/ml of template DNA, Buffer 1X (Promega), 0.2 mmol/L of dNTPS, 0.2 μmol/L of each primer, 1.5 mmol/L of MgCl2, and 1.25 units of GoTaq polymerase (Promega). PCR cycling profile consisted on an initial denaturation at 94°C for 1 min, followed by 30 cycles of denaturation at 94°C for 40 s, annealing at 57–60 °C for 40 s (Table S2) and polymerase extension at 72°C for 40 s, and a final extension at 72°C for 2 min. PCR products were analyzed for length variation using a 3130xl Genetic Analyzer, GeneScan LIZ 600, and Genotyper software (Applied Biosystems, Inc.). The procedure was performed twice for each sample to confirm the results.

#### 2.2.3 mtDNA

A fragment of 530 bp from the mtDNA control region was amplified by PCR using primers THR L15926 (Kocher et al., 1989) and TDKD (Kocher, Conroy, McKaye, & Stauffer, 1993). Each PCR, with a final volume of 50 μl, had the same final concentrations of each reagent used for microsatellite PCRs. The PCR cycling profile was as previously used in Gariboldi et al. (2015). PCR products were purified with a commercial kit (AccuPrep PCR Purification Kit, Bioneer) and sequenced using an ABI 337 Automated DNA Prism Sequencer (Applied Biosystems, Inc.). The procedure was performed twice for each sample to confirm the results.

### 2.3 Microsatellite analyses

For each locus, MICRO‐CHECKER v2.2.3 (Van Oosterhout, Hutchinson, Wills, & Shipley, 2004) was used to check for null alleles, large allele dropout, and scoring errors. The probability of identity (*P*_ID_) for unrelated individuals and for siblings (*P*_ID‐SIB_) for the combined set of loci was assessed using GENALEX v6.5 (Peakall & Smouse, 2012). The *P*_ID_ and *P*_ID‐SIB_ refer to the probability that two unrelated individuals and siblings, respectively, drawn randomly from a population will have the same genotype at multiple loci (Waits, Luikart, & Taberlet, 2001).

Since first‐order relatives can lead to an overestimation of population structure, to avoid this potential bias in our analyses we used the maximum‐likelihood software ML‐RELATE (Kalinowski, Wagner, & Taper, 2006) to identify all potential parent–offspring and full sibling pairs. We first determined the most likely relationship (first order, second order, or unrelated) between individuals within each locality and for the whole sampling area. Then, we performed the specific hypothesis test of relatedness with 2 × 10^5^ simulations of random genotype pairs to analyze whether the putative relationship fit the data better than the alternative relationship (*p *=* *.05): When the putative relationship was parent–offspring, the alternative relationship was full sibling; when the putative relationship was full sibling, the alternative relationship was half sibling. Considering that no parent–offspring relationship was found and none of the putative full sibling pairs first observed (the proportion of putative first‐order related individuals found was <1% in all cases) were supported after the hypothesis test, we included the 69 individuals collected to perform the subsequent analyses.

Possible deviations from Hardy–Weinberg equilibrium (HWE) and linkage disequilibrium (LD) between all locus pairs (1,000 dememorisations, 1,000 batches, and 10,000 iterations per batch) were analyzed using GENEPOP v4.1 (Rousset, 2008). Significance levels (*p *=* *.05) for departure from HWE and LD were corrected for multiple comparisons with a modified false discovery rate (FDR) procedure (hereafter B‐Y) (Benjamini & Yekutieli, 2001) (B‐Y′s *p *=* *.01169). ARLEQUIN v3.5 (Excoffier & Lischer, 2010) was used to estimate the number of alleles and the observed (*H*_O_) and expected heterozygosities (*H*_E_). Allelic richness (AR) as an unbiased measure of the number of alleles adjusted by the sample size was estimated using FSTAT 2.9.5 (Goudet, 2001).

To estimate current migration rate (*m*) among the four sampled localities (NC, CL, MH, and RN), BAYESASS v1.3 (Wilson & Rannala, 2003) was used. The program utilizes genotypes to estimate rates of recent migration among putative populations. As the method is a nonequilibrium Bayesian method, it does not require HWE within populations (Wilson & Rannala, 2003). Five independent runs of 5 × 10^7^ iterations (sampled every 2,000) with 1 × 10^7^ iterations discarded as burn‐in were performed. Since more reliable results are obtained from runs when the number of proposed changes for allele frequencies (*a*), inbreeding coefficient (*f*), and migration rate (*m*) is between 40 and 60% of the total chain length (Wilson & Rannala, 2003), delta values were adjusted as: *m* = 0.5, *a* = 0.6, and *f* = 0.6.

Five tests for evidence of sex‐biased dispersal were conducted using FSTAT 2.9.5 (Goudet, 2001). The first test consisted in comparing population *F*_ST_ values between sexes; allelic frequencies of the dispersal sex should show higher levels of homogeneity across populations than the philopatric sex. The second test compared relatedness (r) between males and females; within populations, r should be greater in the philopatric sex than in the dispersing one. The third test compared *F*_IS_ statistics between sexes; a sex‐biased dispersal should be reflected in a statistically significant higher *F*_IS_ for the dispersal sex (Goudet, Perrin, & Waser, 2002). The other tests consisted in calculating the mean and variance of assignment indices (*mAIC* and *vAIC*, respectively) to determinate the probability of a genotype originating from the population in which the individual was collected (Favre, Balloux, Goudet, & Perrin, 1997). Immigrants to a population, and therefore the dispersal sex, are expected to have lower *mAIC* and higher *vAIC* values than residents (Dubey, Brown, Madsen, & Shine, 2008; Goudet et al., 2002). All tests were performed based on 10,000 permutations.

Population structure among the sampled localities was analyzed through the analysis of molecular variance (AMOVA) using ARLEQUIN v3.5 (Excoffier & Lischer, 2010). Population pairwise *F*_ST_ values (Weir & Cockerham, 1984) were computed using ARLEQUIN V3.5 (Excoffier & Lischer, 2010). Significance levels (*p *=* *.05) were tested using 8,000 nonparametric permutations and corrected with the B‐Y (Benjamini & Yekutieli, 2001) correction.

A Bayesian clustering approach was used to infer putative population differentiation in our data set and to assign individuals to genetic clusters without a prior definition of putative populations, as implemented in STRUCTURE v2.3 (Falush, Stephens, & Pritchard, 2003; Hubisz, Falush, Stephens, & Pritchard, 2009; Pritchard, Stephens, & Donnelly, 2000). Assuming that loci are at HWE and linkage equilibrium within populations, given the number of populations (*K*), individuals in the data set are probabilistically assigned to one or more populations. Since individuals within a population may have mixed ancestry, we used the admixture model. *K* ranging from 1 to 7 was evaluated performing 30 independent Markov chain Monte Carlo (MCMC) runs of 5 × 10^6^ iterations following a burn‐in period of 1 × 10^6^ iterations for each value of *K*. The method of Evanno, Regnaut, and Goudet (2005), which determines the second‐order rate of change of the likelihood function with respect to *K* (∆*K*), was used to determine most likely value of *K* over multiple runs, as implemented in STRUCTURE HARVESTER (Earl & vonHoldt, 2012). In order to assign individuals to clusters, a proportion of membership threshold value of *q *≥* *0.9 (Costa Urrutia et al., 2012; Trigila, Gómez, Cassini, & Túnez, 2016), which indicates that ≥90% of ancestry can be attributed to a specific cluster, was chosen.

In order to test for isolation by distance (IBD), a Mantel test was performed. IBD v3.23 (Jensen, Bohonak, & Kelly, 2005) was used to examine the correlation between *F*_ST_/(1 − *F*_ST_) and the logarithm of the geographic distance between localities. Using a geographic information system (GIS) in ArcGIS software, geographic distances between localities were calculated as the minimum distance by sea between each other. The rejection of the null hypothesis of a flat or negative slope between genetic and geographic distances was used as evidence of IBD.

### 2.4 Mitochondrial DNA analyses

The data set used for mtDNA analysis consisted of 100 sequences: 64 sequences previously published by Gariboldi et al. (2015) for NC, CL, MH, and RN; the 31 sequences previously published by Lázaro et al. (2004) for CL; and five not previously published sequences (one from MH and four from RN). We used CLUSTAL X 2.0.11 (Larkin et al., 2007) to align DNA sequences and to identify polymorphic sites. The mtDNA haplotypes were compared with those previously published for the species (Costa Urrutia et al., 2012; Cunha et al., 2014; Gariboldi et al., 2015; Lázaro et al., 2004; Méndez et al., 2008; Méndez, Rosenbaum, Subramaniam, et al., 2010; Negri, 2011; Secchi, Wang, Murray, Rocha Campos, & White, 1998). ARLEQUIN v3.5 (Excoffier & Lischer, 2010) was used to assess the haplotype (*h*) and nucleotide diversity (π) of the data set.

By using a MCMC approach, migration rates between the sampled locations were estimated with MDIV (Nielsen & Wakeley, 2001). The program estimates the migration rate per gene per generation scaled by the effective population size (*M* = 2*N*_e_ *m*). The finite sites (HKY) model was used. Ten independent runs of 2 × 10^6^ iterations each and a burn‐in of 5 × 10^5^ iterations were performed. For each parameter, likelihood values with the highest posterior probability were accepted as the best estimates.

An AMOVA was performed to analyze the spatial structure among our sampling locations using ARLEQUIN v3.5 (Excoffier & Lischer, 2010). Population pairwise *F*_ST_ statistics were computed in ARLEQUIN v3.5 (Excoffier & Lischer, 2010). Significance levels (*p *=* *.05) were assessed using 8,000 nonparametric permutations and corrected with the B‐Y (Benjamini & Yekutieli, 2001) correction (B‐Y′s *p *=* *.02041).

Similar to microsatellites, a Mantel test based on the mtDNA data set was performed using IBD v2.23 (Jensen et al., 2005).

## 3 Results

### 3.1 Determination of sex

From the analysis of the 69 individuals, we identified molecularly 36 females and 33 males. Female: male ratios were similar through all putative populations (NC 1:1; CL 1:1.1; MH 1:1.3), with the exception of RN (1:0.4).

### 3.2 Microsatellite analyses

The microsatellite loci studied varied with respect to the number of alleles observed, from 7 to 13. Although putative populations had different sample sizes, the mean number of alleles was similar between them (Table 1). Additionally, AR was similar between localities (NC = 6.44 ± 1.03, CL = 6.34 ± 0.94, MH = 6.70 ± 1.49, and RN = 6.27 ± 1.45). The locus with highest number of alleles was FB17, while FB5 was the one with the lowest number. Alleles found exclusively in one of the putative populations (i.e., private alleles) were not observed in CL. The number of private alleles ranged from 1 in NC to 13 in MH (Table 1). No evidence of null alleles or large allelic dropout was found in our data set.

**Table 1:** Genetic diversity values for each locality and loci based on the microsatellite loci data set

		NC	CL	MH	RN
TOTAL	*N*	20	20	14	15
	MA ± *SD*	6.70 ± 1.25	6.70 ± 1.06	6.70 ± 1.49	6.30 ± 1.49
	*H*_O_ ± *SD*	0.64 ± 0.01	0.70 ± 0.02	0.73 ± 0.03	0.73 ± 0.02
	*H*_E_ ± *SD*	0.79 ± 0.02	0.77 ± 0.02	0.79 ± 0.01	0.79 ± 0.01
MK8	NA	7	8	8	7
	PA	0	0	2	0
	*H*_0_	0.6	0.75	0.85	0.73
	*H*_E_	0.69	0.78	0.8	0.78
D22	NA	6	6	6	6
	PA	0	0	0	0
	*H*_0_	0.60	0.60	0.71	0.73
	*H*_E_	0.79	0.75	0.8	0.84
FB5	NA	5	7	7	6
	PA	0	0	0	0
	*H*_0_	0.70	0.8	0.79	0.60
	*H*_E_	0.77	0.83	0.84	0.79
EV14	NA	6	5	7	6
	PA	1	0	4	1
	*H*_0_	0.55	0.65	0.79	0.60
	*H*_E_	0.78	0.65	0.85	0.80
EV5	NA	8	6	8	5
	PA	0	0	1	1
	*H*_0_	0.65	0.65	0.79	0.8
	*H*_E_	0.85	0.82	0.88	0.76
FB2	NA	8	8	5	7
	PA	0	0	0	1
	*H*_0_	0.65	0.60	0.71	0.73
	*H*_E_	0.83	0.83	0.81	0.82
MK6	NA	6	6	7	6
	PA	0	0	2	2
	*H*_0_	0.65	0.65	0.64	0.73
	*H*_E_	0.82	0.79	0.83	0.85
FB17	NA	9	8	9	10
	PA	0	0	1	1
	*H*_0_	0.70	0.80	0.79	0.80
	*H*_E_	0.88	0.84	0.85	0.89
MK5	NA	6	7	6	5
	PA	0	0	2	1
	*H*_0_	0.65	0.75	0.64	0.80
	*H*_E_	0.82	0.80	0.85	0.8
EV94	NA	6	6	4	5
	PA	0	0	1	1
	*H*_0_	0.65	0.70	0.57	0.73
	*H*_E_	0.79	0.76	0.71	0.79

*N*, number of individuals; MA, mean number of alleles; *H*_O_, observed heterozygosity; *H*_E_, expected heterozygosity; *SD*, standard deviation; NA, number of alleles; PA, number of private alleles. NC, Necochea; CL, Claromecó; MH, Monte Hermoso; RN, Río Negro.

The analyzed set of microsatellites was powerful enough to discriminate between individuals within putative populations. Theoretical *P*_ID_ and *P*_ID‐SIB_ values within populations varied between 5.8 × 10^−12^ − 1.9 × 10^−11^ and 5.4 × 10^−5^ − 7.2 × 10^−5^, respectively.

CL, MH, and RN showed similar levels of heterozygosity, and the mean *H*_O_ ranged from 0.64 in NC to 0.73 in MH and RN (Table 1). We did not find significant deviation from HWE at any of the analyzed microsatellite loci after B‐Y correction (all *p *ϣ.05). Additionally, we did not find significant LD between all pair of microsatellite loci within putative populations after B‐Y correction (all *p *ϣ.05). Therefore, we considered that all loci were genetically independent.

The global test of genetic differentiation was significant (*F*_ST_ = 0.050; *p *<* *10^−5^); the greatest source of variation was found within putative populations (95.01%). All pairwise comparisons were statistically significant, except the one between CL and NC (Table 2).

**Table 2:** Pairwise genetic differentiation between putative populations for the microsatellite loci and the mtDNA control region

	Microsatellite				mtDNA			
	NC	CL	MH	RN	NC	CL	MH	RN
NC	0.00	0.77	<10^−5^	<10^−5^	0.00	0.58	0.01	0.04
CL	−0.00	0.00	<10^−5^	<10^−5^	−0.01	0.00	0.00	0.11
MH	**0.05**	**0.07**	0.000	<10^−5^	**0.11**	**0.10**	0.00	<10^−5^
RN	**0.05**	**0.06**	**0.09**	0.00	0.06	0.03	**0.25**	0.00

NC, Necochea; CL, Claromecó; MH, Monte Hermoso; RN, Río Negro. *F*_ST_ values are shown below the diagonal and *p*‐values are shown above the diagonal.

Significant values at *p *<* *.01 (for microsatellite loci) and *p *<* *.02 (for mtDNA) are shown in bold.

**Figure 2:**
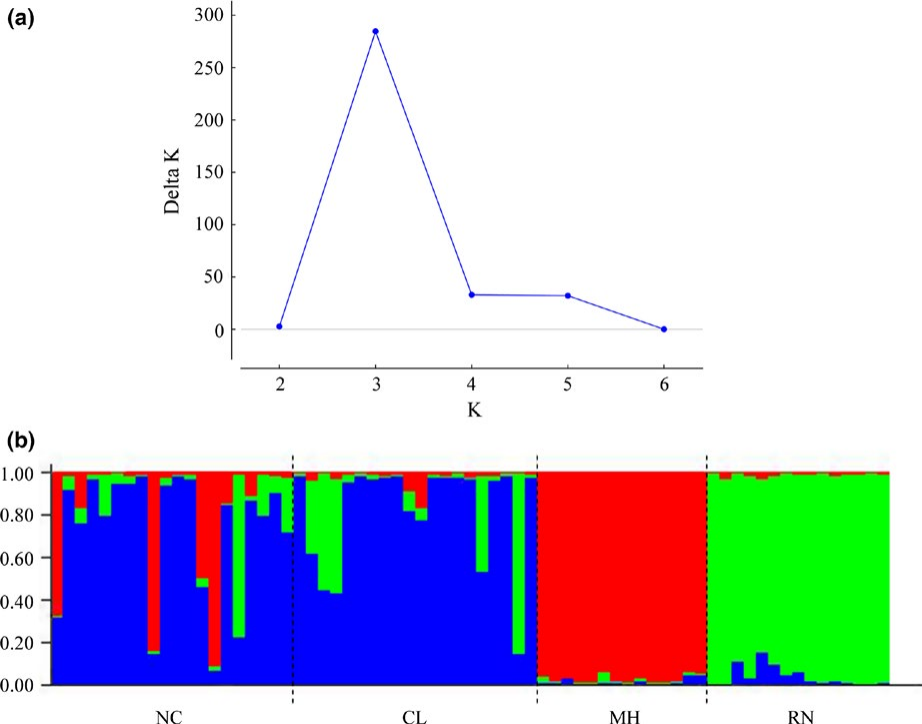
Bayesian clustering assignment based on the microsatellite loci data set. (a) Delta *K* values obtained by the Evanno's method (Evanno et al., 2005), where the modal value of the distribution is considered as the highest level of structuring. (b) Individual assignment to each of the three clusters, where each vertical column represents an individual and the proportion of each color indicates the proportion of ancestry. Detailed geographic origin of the samples is given below the graphic. NC, Necochea; CL, Claromecó; MH, Monte Hermoso; RN, Río Negro

In accordance with the AMOVA results, the Bayesian model‐based clustering method showed a clear population subdivision. Considering a range between 1 and 7 populations, the analysis suggested the existence of three populations genetically differentiated (Figure 2). Assuming *K* = 3, almost all individuals from RN and MH were assigned to their respective cluster (except for two individuals from RN with *q* = 0.89 and *q* = 0.82). In the case of CL‐NC, twenty‐two individuals (55%) were assigned to a common cluster.

Estimated migration rates were low between all locations, except from CL to NC (Table 3). The latter suggests unidirectional migration between both locations. Multiple runs showed consistent results.

**Table 3:** Estimates of migration rate between putative populations based on the microsatellite loci data set

[RN] [RN]: 0.90 (0.04)	[RN] [CL]: 0.09 (0.05)	[RN] [MH]: 0.01 (0.01)	[RN] [NC]: 0.05 (0.04)
[CL] [RN]: 0.01 (0.01)	[CL] [CL]: 0.72 (0.07)	[CL] [MH]: 0.01 (0.01)	[CL] [NC]: 0.26 (0.07)
[MH] [RN]: 0.03 (0.02)	[MH] [CL]: 0.04 (0.04)	[MH] [MH]: 0.90 (0.06)	[MH] [NC]: 0.03 (0.03)
[NC] [RN]: 0.02 (0.01)	[NC] [CL]: 0.05 (0.05)	[NC] [MH]: 0.02 (0.02)	[NC] [NC]: 0.91 (0.08)

NC, Necochea; CL, Claromecó; MH, Monte Hermoso; RN, Río Negro.

Mean (standard deviation) posterior distributions for each migration rate among franciscana dolphin locations are shown. Values between the same location represent the proportion of individuals derived from the source population (nonmigrant) each generation.

**Figure 3:**
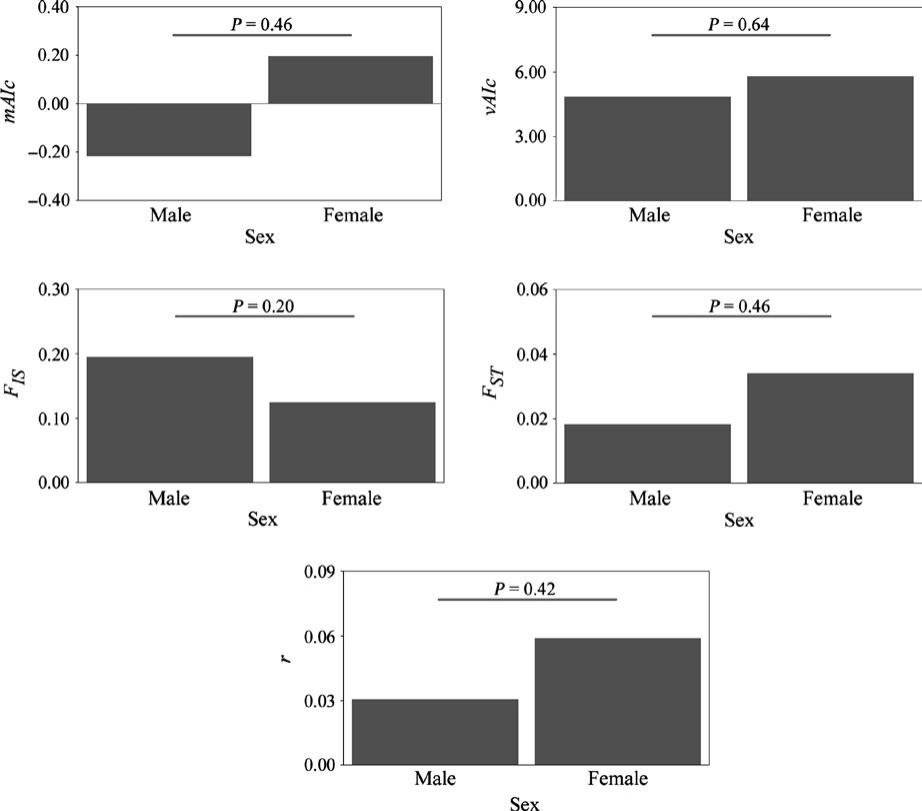
Sex‐biased dispersal analyses: mean assignment index (*mAIc*), variance of the assignment index (*vAIc*), *F*_ST_, *F*_IS,_ and relatedness (r). Correspondent *p*‐values are shown above each test

The entire tests conducted to detect sex‐biased dispersal were not statistically significant (Figure 3). Therefore, our results do not support the hypothesis that females nor males are more dispersive than the other sex.

**Figure 4:**
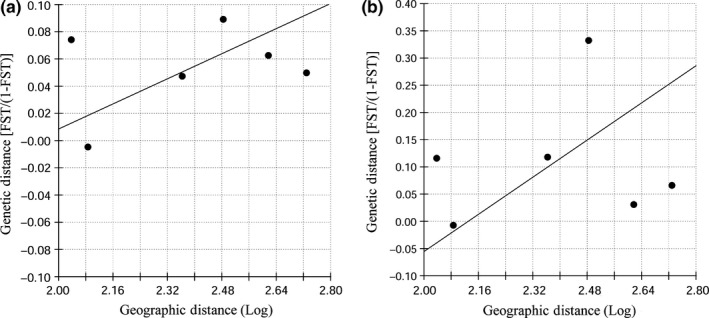
Mantel test for IBD based on the microsatellite loci (a) and the mtDNA control region (b) data sets

A positive relation was observed between the geographic and the genetic distance between putative populations (Figure 4a). However, the correlation was not statistically significant (r = 0.325; *p *=* *.333).

### 3.3 mtDNA analyses

From the analysis of the mtDNA control region of the new samples collected for this study, no new haplotypes were found. Instead, three haplotypes previously described in Lázaro et al. (2004), L5 (*n* = 2), L15 (*n* = 1), and L22 (*n* = 1), were found in RN and another haplotype previously described in Gariboldi et al. (2015), G4 (*n* = 1), was found in MH. When combining our samples with those previously published (Gariboldi et al., 2015; Lázaro et al., 2004), a total of 23 haplotypes were found between NC and RN (Table 4). MH showed the lowest haplotype and nucleotide diversity values, and NC the highest (Table 4).

**Table 4:** Genetic diversity indices for each locality based on the mtDNA control region data set

	*N*	*n*	*H*	π
NC	20	9	0.90 + −0.04	0.01 + −0.01
CL	51	16	0.84 + −0.04	0.01 + −0.01
MH	14	6	0.68 + −0.13	0.01 + −0.01
RN	15	5	0.73 + −0.09	0.01 + −0.01
Total	100	23		

*N*, sample size; *n*, number of haplotypes; *H*, haplotype diversity; π, nucleotide diversity. NC, Necochea; CL, Claromecó; MH, Monte Hermoso; RN, Río Negro.

The AMOVA showed a significant global genetic differentiation between putative populations (*F*_ST _= 0.081; *p *=* *.003). As for microsatellites, the greatest source of variation was found within putative populations (91.92%). Results of the pairwise comparisons showed no significant differences between NC and CL, NC and RN, or between CL and RN; all other pairwise comparisons were statistically significant (Table 2).

In accordance with the mtDNA population structure results, higher levels of gene flow were observed between NC and CL (*m* = 32.48) and, to a lesser extent, between CL and RN (*m* = 11.04). All other population pairs showed values lower than *m* = 2.28.

Similar to microsatellite loci data set, mtDNA data showed a positive but not significant correlation between the geographic and genetic distances between putative populations (r = 0.128; *p *=* *.373) (Figure 4b).

## 4 Discussion

Differentiation among groups of individuals is a fundamental topic in population genetics. In this sense, the use of multiple molecular markers may provide valuable information about the processes that shape the population structure of a species. In this study, we have extended the knowledge of population structure of the franciscana dolphin, the most threatened small cetacean in the southwestern Atlantic Ocean (Secchi, 2010; Secchi et al., 2003). Maternally inherited mtDNA control region sequences and biparentally inherited microsatellite loci‐based analyses were performed in order to study the patterns of genetic structuring in the southernmost edge of the species geographic range.

### 4.1 Genetic diversity

Overall, similar levels of genetic variation for both the microsatellite and mtDNA data were observed between localities in this study (Tables 1 and 4), and these were comparable with those previously found in the FMA IV (Méndez et al., 2008; Méndez, Rosenbaum, Subramaniam, et al., 2010). We found that MH and RN presented several private alleles, in comparison with NC and CL (Table 1), and that these were found in all, but one case, at the edges of the allele sizes (data not shown). This was an expected result since private alleles at a locus tend to be found in the edges of the allele size distribution, that is, commonly they have very long or short repeat lengths with respect to the other alleles at that locus, and this probability is positively correlated with the pairwise *F*_ST_ estimates between populations (Szpiech & Rosenberg, 2011).

Moreover, it is important to emphasize that among our microsatellite data set, we did not find null alleles that may lead to an overestimation of population genetic differentiation by reducing gene diversity (Chapuis & Estoup, 2007; Putman & Carbone, 2014).

### 4.2 Sex‐biased dispersal

Regarding sex‐biased dispersal, although mammals often exhibit a pattern of male‐biased dispersal and female philopatry (Greenwood, 1980) and sex‐biased dispersal is common in other cetacean species (e.g., Escorza Treviño & Dizon, 2000; Hoelzel et al., 2007; Krützen, Sherwin, Berggren, & Gales, 2004; Möller & Beheregaray, 2004), our results did not detect a bias in dispersal between the sexes (Figure 3). Although this result should be regarded with caution since the ability of the tests performed to detect the bias in dispersal is limited mainly due to the lack of extreme bias in dispersal, the low to moderate dispersal estimates, and the number of loci and samples analyzed (Goudet et al., 2002), previous franciscana dolphin studies also found a lack of sex‐biased dispersal (Costa Urrutia et al., 2012; Méndez et al., 2008), supporting our findings.

### 4.3 Population structure and gene flow

The analyses based on mtDNA and microsatellite loci showed evidence of population genetic structure at the southernmost portion of the franciscanas’ range. In general, the estimates of genetic divergence were generally higher for the mtDNA than for the microsatellite data set (Table 2). This was expected based on the lack of sex‐biased dispersal (Figure 3) and the fact that the mtDNA reduces the effective population size to one‐fourth that of nuclear genes due to its matrilineal inheritance pattern and haploid nature (Birky, Maruyama, & Fuerst, 1983; DeSalle, Templeton, Mori, Pletscher, & Johnston, 1987) and therefore changes in population allele frequencies may accumulate faster in this marker than in microsatellites.

Nevertheless, only the microsatellite data set revealed a significant differentiation between RN and all other localities (Table 2). Considering these results, three plausible and nonexclusionary hypotheses can be made to explain the contrasting pattern between molecular markers observed in this study. First, mtDNA and microsatellite loci may be showing different temporal genetic patterns due to differences in their rate and pattern in mutation (Balloux & Lugon Moulin, 2002; Wan, Wu, Fujihara, & Fang, 2004). Second, based on the allele frequency distributions and the ratio *R* = *F*_ST,organelle_/*F*_ST,nuclear_, the differences may be reflecting the use of a single organelle marker (mtDNA) versus 10 nuclear markers (microsatellite loci) (Larsson, Charlier, Laikre, & Ryman, 2009). Finally, based on the migration rates estimates (Table 3 and mtDNA results) and since under selective pressures dispersal strategies can evolve (Lawson Handley & Perrin, 2007), the contrasting pattern between molecular markers may be reflecting a greater past female‐mediated gene flow than the one mediated by males.

Among putative populations, migration is a central driving force in evolution that reduces local adaptation (Abdo, Crandall, & Joyce, 2004; Meirmans, 2014). In accordance with a previous mtDNA control region analysis (Gariboldi et al., 2015), high rates of gene flow (Table 3 and mtDNA results) and a lack of genetic differentiation between CL and NC were observed based on the microsatellite loci and mtDNA analyses (Table 2), suggesting that individuals from both localities form a panmictic population. This may be due to close geographic proximity between localities which may entail similar resource use, as it was previously suggested for this species (Costa Urrutia et al., 2012; Gariboldi et al., 2015; Méndez et al., 2008; Méndez, Rosenbaum, Subramaniam, et al., 2010) and other cetaceans (Fullard et al., 2000; Hoelzel et al., 2007; Natoli et al., 2005). In fact, individuals from NC and CL were found to fed primary on *Loligo sanpaulensis* (Paso Viola, 2014; Paso Viola et al., 2014).

In the case of MH, in agreement with its previously reported separation as a genetically different population (Gariboldi et al., 2015), both microsatellite and mtDNA data revealed a significant differentiation between MH and all the other analyzed localities (Table 2). Based on the close relative geographic proximity between MH and CL and the low levels of gene flow between each other (Table 3 and mtDNA results), the observed genetic differentiation between the two localities may be due to different resource specializations. Prey distribution and abundance are thought to influence the occurrence and distribution of cetaceans (Hastie, Wilson, Wilson, Parsons, & Thompson, 2004) and may lead to intraspecific differentiation by means of resource specialization (Hoelzel, 1998), as it has been previously suggested for other species of dolphins (e.g., Bilgmann, Möller, Harcourt, Gales, & Beheregaray, 2008; Bilgmann, Möller, Harcourt, Gibbs, & Beheregaray, 2007; Möller, Wiszniewski, Allen, & Beheragaray, 2007; Sellas et al., 2005). Since individuals from MH and CL were found to have different diet preferences—individuals from CL feed primary on *Loligo sanpaulensis* and MH majority preys are *Cynoscion guatucupa* and *Artemesia longinaris* (Paso Viola, 2014; Paso Viola et al., 2014)—it is feasible to consider that genetic differentiation between these geographically close localities could have been promoted and maintained over time by prey specialization. In fact, Gariboldi et al. (2015) recently proposed that, after its colonization by few maternal lineages, the maintenance in MH of a reduced mtDNA diversity and a relative constant size over time is due to low levels of gene flow between this and other geographically close localities likely promoted by resource specializations. In the case of MH and the other localities, genetic differences may be related to a nonrandom mating between individuals due to the species’ relative small home ranges (Bordino et al., 2008; Wells, Bordino, & Douglas, 2013) and the geographic distance between localities, as previously suggested (Gariboldi et al., 2015).

Additionally, although the genetic population structure of many species may be characterized by a pattern of IBD (e.g., Ansmann, Parra, Lanyon, & Seddon, 2012; Hoelzel et al., 2007; Natoli et al., 2005), we did not observe a significant correlation between genetic differentiation and geographic distance among populations (Figure 4). If equilibrium between the loss of alleles due to genetic drift and their replacement by gene flow between populations exists, the genetic distance between populations will increase with geographic distance due to the changing influence of gene flow and genetic drift as populations become more or less geographically separated (Hutchison & Templeton, 1999). However, this pattern may be affected by time and restrictions in dispersal within an area. Therefore, if time since colonization of a given area is relatively short and gene flow remains strong relative to genetic drift, a pattern reflecting panmixia would persist, as it may be reflecting NC and CL. If lower levels of gene flow exist, a stronger influence of genetic drift through time will occur, and eventually higher genetic differentiation will be observed, as it may be the case of RN. In the case of MH, changes in environmental conditions and resource specializations may conduct to a stronger influence of genetic drift than gene flow, despite the closer geographic distance between this population and others. These cases for deviation from equilibrium gene flow/genetic drift may explain the lack of evidence of IBD in our study area.

Bayesian clustering analyses such as STRUCTURE (Pritchard et al., 2000) can be used to evaluate breaks in allele frequencies, but can overestimate genetic structure in data sets that are characterized by IBD (Frantz, Cellina, Krier, Schley, & Burke, 2009). However, the use of ∆*K* (Evanno et al., 2005) that may reduce the number of artificial clusters when compared with other Bayesian clustering methods (Frantz et al., 2009), the lack of strong patterns of IBD among our study area (Figure 2) (Frantz et al., 2009; Meirmans, 2012), and the correspondence with the genetic pattern obtained through the AMOVA and pairwise values of genetic differentiation (Table 2) further suggest the existence of three genetic clusters or populations in our study area: NC/CL, MH, and RN.

Finally, based on mtDNA and microsatellite loci data, Méndez, Rosenbaum, Subramaniam, et al. (2010) reported that their BASW location, near RN, did not differ from their BAS location (named as NC and CL in this study). Based on the published information of that study, we were not able to compare our results with neither BASW nor BAS. However, BAS location was composed by samples from NC and the 31 mtDNA control region sequences from CL reported by Lázaro et al. (2004) and included in our study. Therefore, it is possible that NC/CL would not differ from BASW and also that RN would comprise a population genetically distinct from NC/CL/BASW.

### 4.4 Management and conservation implications

In this study, we have reported a fine‐scale genetic structure for the franciscana dolphin over the southernmost portion of the species range, uncovering a new genetic distinct population, RN. Based on these results and previous studies (Gariboldi et al., 2015; Méndez et al., 2008; Méndez, Rosenbaum, Subramaniam, et al., 2010), five populations are found within Argentina: SW/SS, CSA/BAE, MH, NC/CL/BASW, and RN. Furthermore, our study highlights the need to perform multilocus analyses to identify genetically distinct populations since allele frequency distributions, rates of gene flow, mutation rates, and effective population sizes may affect the statistical power of molecular markers (Larsson et al., 2009) and lead to a misinterpretation of the true genetic relationships among populations.

Incidental annual mortality of the franciscana dolphin in Argentina represents up to 2%–5% of its abundance in the area (e.g., Cappozzo et al., 2007; Crespo et al., 2010; Negri et al., 2012) which, according to the International Whaling Commission Scientific Committee (Donovan & Bjørge, 1995), may not be sustainable over time. Additionally, considering our results, MH may have become isolated from geographically close populations due to specializations over resources, whereas RN might have diverged recently from CL‐NC due to the geographic distance between them. Consequently, if these populations were severely impacted by certain factors, such as high incidental bycatch, genetic depletion may not be able to be counteracted by gene flow. Therefore, reformulating Secchi′s et al. (2003) FMAs’ division is necessary. The development of conservation and management plans should take into account each genetically distinct population found in Argentina as different management units (sensu Moritz, 1994, 2002), considering the mechanisms that may have prompted genetic differentiation between them, as well. However, conservation and management strategies need to be developed upon reliable demographic data. In this regard, the abundance of the species along the Argentina coast was estimated in a single study (Crespo et al., 2010). Additionally, although incidental mortality rates have been assessed previously (e.g., Cappozzo et al., 2007; Crespo et al., 2010; Negri et al., 2012), there are some areas, such as RN, with no information, whereas information needs to be updated in others. Therefore, it is crucial to first carry out demographic studies within each management unit.

## Conflict of interest

None declared.

## Data accessibility

Relevant data are within the paper and its Supporting Information file.
