# Supplementary material for: Patterns of population structure at microsatellite and mitochondrial DNA markers in the franciscana dolphin (Pontoporia blainvillei)
Source: Ecol Evol. 2016 Nov 17;6(24):8764–76. doi: 10.1002/ece3.2596 (PMC5192793; doi:10.1002/ece3.2596)
Supplement: Supplementary file 1 [file ECE3-6-8764-s001.docx]

Supporting Information

**Table S1. PCR primers for sex determination.**

| **Primer** | **Concentration** | **Target** | **Reference** |
| --- | --- | --- | --- |
| ZFX0582F | 0.2 uM | ZFX | Bérube & Palsbøll (1996) |
| ZFX0923R | 0.2 uM | ZFX | Bérube & Palsbøll (1996) |
| PMSRYF | 0.2 uM | SRY | Richard *et al.* (1994) |
| TtSRYR | 0.4 uM | SRY | Rosel (2003) |

**Table S2. Microsatellite loci analyzed, repeat motif and their annealing temperatures (TA).**

| **Locus** | **Repeat motif** | **TA (°C)** | **Reference** |
| --- | --- | --- | --- |
| MK5 | (GT)_n_ and (TA)_n_ | 59 | Krützen *et al.* (2001) |
| MK6 | (GT)_n_ | 59 | Krützen *et al.* (2001) |
| MK8 | (CA)_n_ | 59 | Krützen *et al.* (2001) |
| EV5Pm | (GC)_n_ and (GT)_n_ | 55 | Valsecchi & Amos (1996) |
| EV14Pm | (GT)_n_ | 55 | Valsecchi & Amos (1996) |
| EV94Mn | (TC)_n_ and (AC)_n_ | 55 | Valsecchi & Amos (1996) |
| D22 | (CA)_n_ | 59 | Shinohara *et al.* (1997) |
| DlrFB2 | (TG)_n_ | 55 | Buchanan *et al.* (1996) |
| DlrFB5 | (GT)_n_ | 59 | Buchanan *et al.* (1996) |
| DlrFB17 | (GT)_n_ | 60 | Buchanan *et al.* (1996) |
